# Mid- and long-term efficacy of surgical treatment of Vancouver B2 and B3 periprosthetic femoral fractures

**DOI:** 10.1186/s12893-020-00891-9

**Published:** 2020-10-07

**Authors:** Jian-Ning Sun, Yu Zhang, Ye Zhang, Jia-Ming Zhang, Xiang-Yang Chen, Shuo Feng

**Affiliations:** 1 Department of Orthopedic Surgery, Affiliated Hospital of Xuzhou Medical University, 99 Huaihai Road, Xuzhou, 221002 Jiangsu China; 2 Department of Orthopedic Surgery, The Third Hospital of Hebei Medical University, 139 Ziqiang Road, Shijiazhuang, 050000 Hebei China

**Keywords:** Vancouver B2/B3, Periprosthetic femoral fractures, Hip arthroplasty

## Abstract

**Background:**

The incidence of fractures around the femoral prosthesis among patients undergoing hip arthroplasty is increasing and has become the third leading cause of hip revision. While numerous methods for the surgical treatment of periprosthetic femoral fractures (PFFs) have been proposed, only few reports have examined the long-term efficacy of surgical treatment. This study aims to examine the mid-and long-term efficacy of surgical treatment among patients with Vancouver B2 and B3 PFFs.

**Methods:**

This retrospective study evaluated the surgical outcomes of patients with Vancouver B2 and B3 PFFs between 2007 and 2011. The minimum follow-up time was eight years. Fracture healing, prosthesis stability, complications, patient quality of life SF-36 score, and survival rate were evaluated during the follow-up assessments.

**Results:**

A total of 83 patients were included and had an average follow-up period of 120.3 months. Among these patients, 69 were classified as Vancouver B2 and were treated with a distal fixation stem, whereas 14 cases were classified as Vancouver B3 and were treated with modular femoral prosthesis by using a proximal femoral allograft technique. A total of 15 patients underwent secondary revision surgery, and prosthesis dislocation was identified as the main cause of secondary revision. 80 (96.4%) cases of fractures were clinically healed. The mortality rate in the first year after surgery was 8.4% (7/83). The overall 5-year Kaplan–Meier survival rate for these patients was 75.9%. Meanwhile, the 5-year Kaplan–Meier survival rate for the implants was 86.9%. The final follow-up SF-36 score of the patients was 48.3 ± 9.8.

**Conclusions:**

Patients with Vancouver B2 and B3 PFFs show high mortality in the first year after their surgery, and the Kaplan–Meier analysis results showed that such mortality tends to plateau after 5 years. Prosthesis dislocation was identified as the primary cause of secondary revision.

## Background

After aseptic loosening and infection, periprosthetic femoral fractures (PFFs) have been identified as the third leading cause of revision, with incidence rates ranging from 0.1 to 2.1% [1, 2]. Incidence of PFFs continues to increase every year along with the continuous aging of the population and the increasing frequency of primary hip replacements [3, 4]. Data from the Swedish National Arthroplasty Registry revealed that revision surgery caused by PFFs account for approximately 9.3 to 14.7% of all hip revision surgeries, while that the cases of re-fractures after revision were approximately 5 times higher than those following primary hip replacements [5].

PFFs have many risk factors, including trauma, age, gender, osteoporosis, and prosthesis loosening. The most common cause of PFFs among patients is mild trauma like ground level falls, which account for approximately 75 to 84% of all reported cases [5]. PFFs may also occur during surgery depending on the choice of revision prosthesis. To achieve initial stability, uncemented fixation requires tight compression during operation. However, uncemented fixation entails a higher fracture risk compared with cement fixation.

Patients with PFFs have a high mortality rate and are exposed to several complications [6, 7]. For the patient, to withstand traumas caused by fractures and revision surgery, whereas for doctors, surgery for PFFs is difficult and time consuming.

The Vancouver classification [8] is the most widely used classification for PFFs that not only identifies the location of fractures but also considers prosthesis stability and bone defects, thereby providing valuable guidance to clinical treatment plans.. Among the Vancouver PFF classes, the Vancouver B-type fracture has the highest incidence rate, with Vancouver B2 and B3 fractures being the most difficult to treat due to prosthesis loosening [9]. However, only few long-term reports on the surgical treatment of Vancouver B2 and B3 fractures have been published. To fill this gap, this study investigates the fracture healing, prosthesis stability, incidence of complications, quality of life, and survival of patients with Vancouver B2 and B3 fractures.

## Methods

### Patient inclusion and data collection

The surgical data collected from Vancouver B2 and B3 patients who were admitted to the affiliated hospital of Xuzhou Medical University and the Third Hospital of Hebei Medical University from 2007 to 2011 were retrospectively analyzed. A total of 97 patients were included in this study, and 14 of them were excluded due to incomplete follow-up data or loss of contact. 83 patients were eventually included in the study. A total of 83 patients were included in the study. Among these patients, 13 had fractures after a revision procedure, whereas 52 developed fractures following a primary total hip replacement. These fractures occurred between 4 and 133 months (average of 80 ± 26.6 months) after primary operation. Among the examined cases, a total of 18 fractures occurred during surgery, of which 12 occurred during the stem-fitting process in primary hip replacement. The proximal bone does not provide initial stability for the short stem prosthesis, we replaced short stem with a long stem. 6 occurred during revision surgery (4 fractures occurred during the stem-fitting process, 2 cases occurred during the removal of cement). A total of 65 cases before revision surgery, 46 were caused by mild trauma (such as fall off a stool or fall on flat ground),13 were due to car accidents, and 6 showed no clear injury (Table 1). Those patients with incomplete follow-up data, femoral malformation, fractures resulting from tumors or long-term use of hormones, acetabular prosthesis fractures, and previous cases of periprosthetic infection were excluded from the study. Fracture classification is based on Vancouver classification standards. The controversial part is classified according to the preoperative X-ray and the specific situation of the fracture during the operation.

**Table 1:** Vancouver classification of fractures, and time interval between index arthroplasty and fracture

Vancouver Category	Primary-Replacement Group	Revision Group	Intraoperative fracture Group	Time interval between index arthroplasty and fracture	Total
B2	44(64%)	10(14%)	15(22%)	78.6 ± 25.3 (range,14–133)	69
B3	8(58%)	3(21%)	3(21%)	88.3 ± 32.7 (range,4–121)	14

### Surgical technique

All surgeries were performed at the tertiary referral center. The arthroplasty surgeons received the same qualified technical training in PFF treatment. Each chief physician had extensive experience in hip revision surgery and fracture fixation. Those patients with preoperative medical diseases were treated by relevant physicians, and the anesthesiologists performed the preoperative risk assessment.

**Fig. 1:**
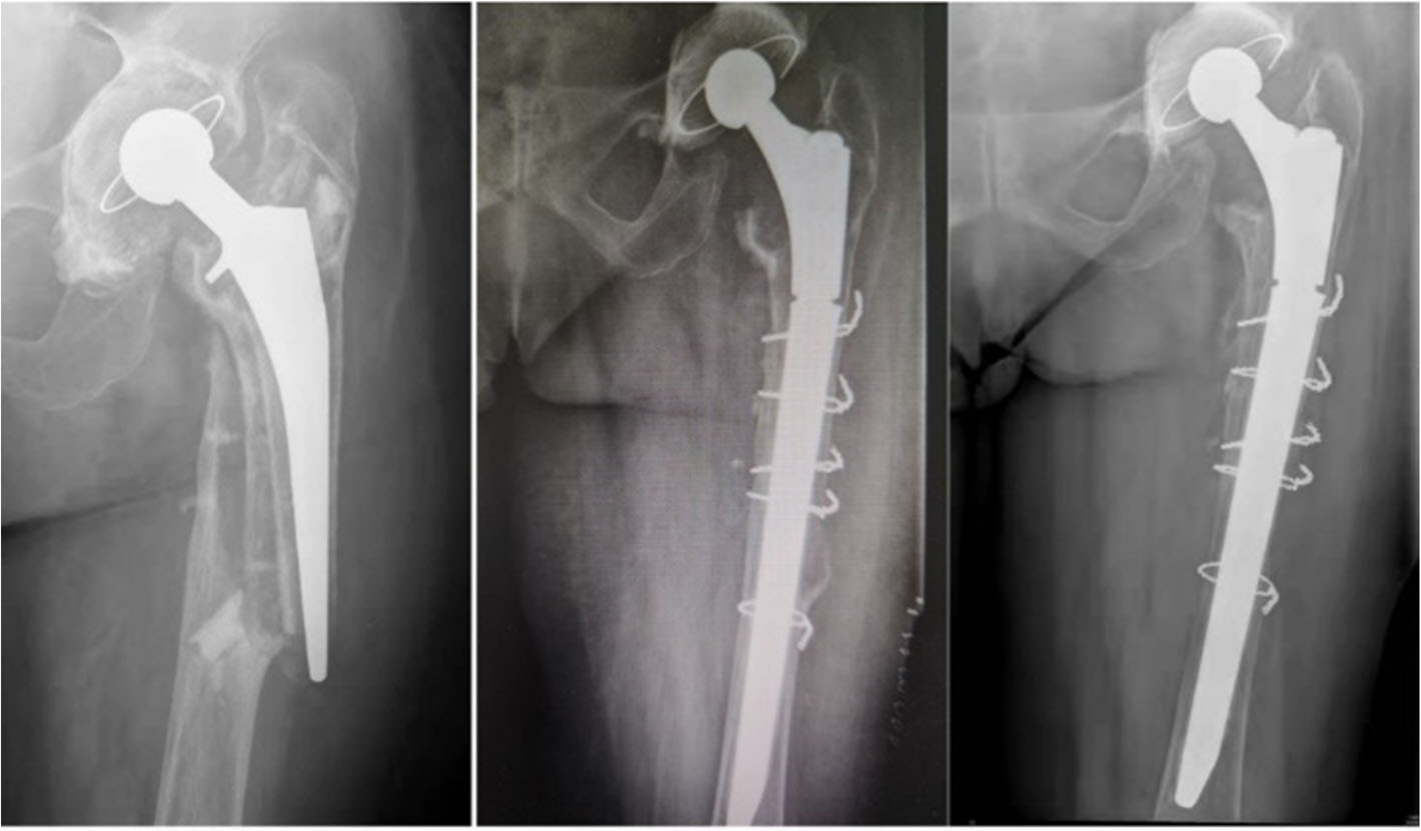
Cemented stem, fall, B2 fracture. Exchange to a modular stem. After 5 years of follow-up, no subsidence or loosening of the implant

**Fig. 2:**
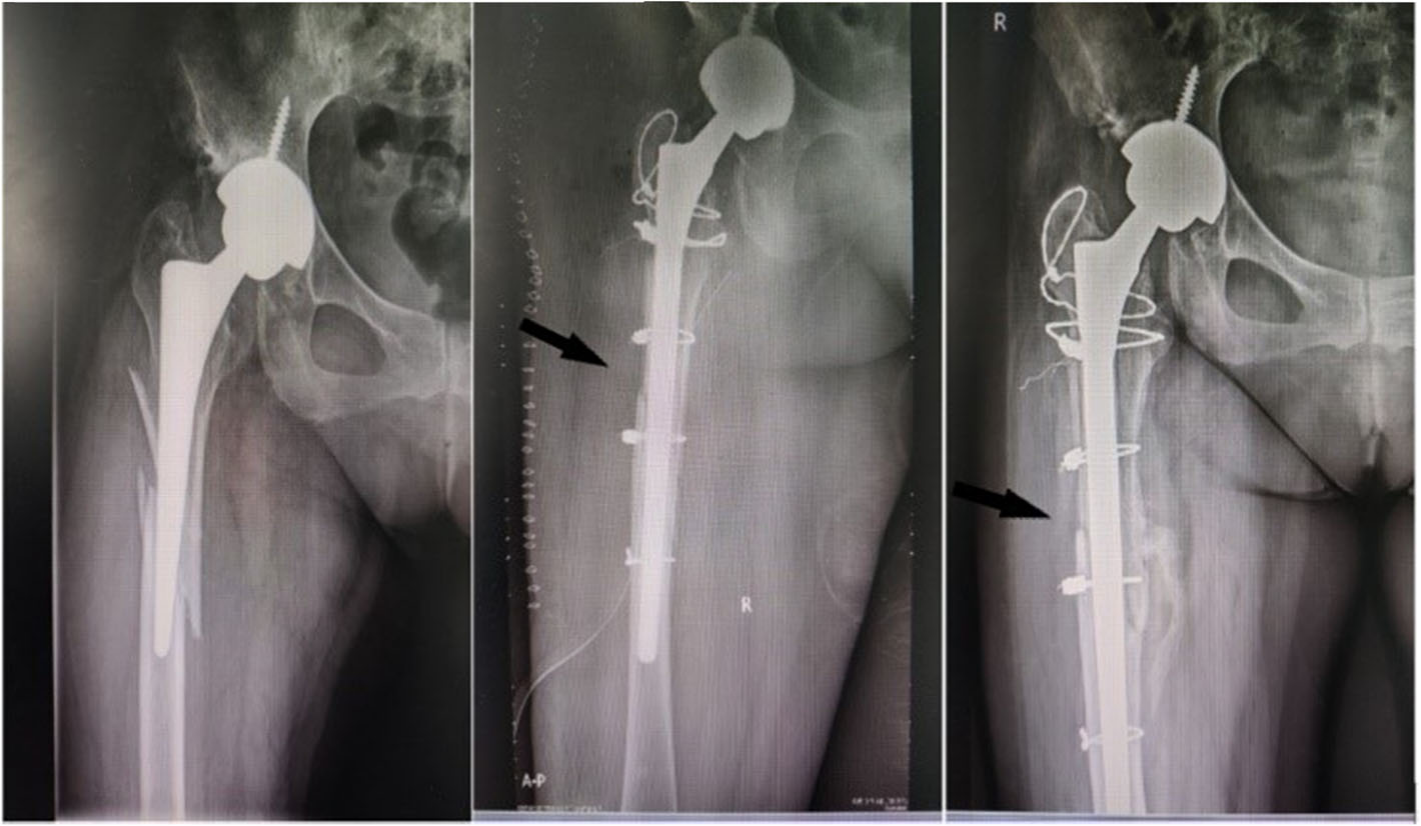
Uncemented stem, traffic accident, B2 fracture. Exchange to a nonmodular stem. After 1 years of follow-up, fracture unhealed

All patients were given general anesthesia and placed in a lateral position. The posterior lateral approach was applied in the surgery. The loosening of the femoral stem prosthesis during surgery was examined along with femoral bone mass. Extended trochanteric osteotomy was performed for those patients having difficulties in removing their original prosthesis. First, the loose prosthesis and any residual cement were removed. Second, the medullary cavity was expanded distally, and the appropriate implant was selected. Third, two to three lines of steel wire or cable were used to fix the osteotomy and fracture pieces around the proximal prosthesis, and the soft tissue around the fracture was retained (Figs. 1 and 2). For those patients with severe femoral cortical thinning, the femur was reconstructed by using structural cortical bone grafts. Methods of intraoperative bone grafting include apply ICA (impacted cancellous allograft) alone and the combination of ICA + CSA (cortical strut allograft). For mild to moderate bone defects, adequately intramedullary ICA was performed to restore the bone store, adequately intramedullary impacted cancellous allograft was performed to restore the bone stock. But when dealing with severe bone defects, the CSA was employed in addition. Choose the right strut allograft which best matched the host bone size radiographically. The strut allograft was cut to fit the shape of the host femur and allowed at least 5 cm exceeding the distal end of the fracture line. The cancellous allografts were used to restore the intramedullary bone defects and fill the space of the cortical strut to the host bone. Fluoroscopy was used during the operation to observe the stability of prosthesis and the reduction in fracture. The wounds were stitched, and a drainage tube was placed.

### Post-operative treatment

There is no unified postoperative mobilization regime for the patients underwent revision surgery. The rehabilitation plan must be individualized. The formulation of the rehabilitation plan must consider the patient’s age, bone condition, comorbidities, soft tissue condition, implant type and stability. Although the revision surgery may compromise the patient’s early mobilization, every effort must be made to get the patient out of bed as soon as possible. For patients who have good bone and soft tissue conditions and without bone graft, we attempted similar mobilesation strategy to that of normal revision THA. On the 5–7 days after surgery, the patient used a walker to assist walking with the affected limb without weight bearing. The affected limb gradually bears weight 3 months after the surgery.

The patients were evaluated after surgery. The first review took place six weeks after surgery and was conducted every three months thereafter during the first year. AP pelvic and lateral radiographs of the affected hip were used in the imaging evaluation. The X-ray captured during the sixth week was used as benchmark in evaluating the position of prosthesis. The Engh [10] standard was used to evaluate the loosening of prosthesis. Loosening is diagnosed when greater than 2 mm of radiolucency are discovered.

The termination time for implant survival was defined as those stems that required revision surgery for any reason. Serious complications included prosthesis loosening, non-union, infection, dislocation, and secondary fracture during follow-up. SF-36 was used to evaluate the quality of life of patients without dementia. SF-36 was followed up every year after revision surgery. For those patients who died after surgery, their relatives were contacted to record the time and cause of their deaths.

### Statistical method

Statistical analyses were performed by using SPSS 19.0 (Chicago, IL, USA), the patient and implant survival was assessed by using the Kaplan–Meier survival curve, and the survival curves were compared by performing a log-rank (Mantel–Cox) test. Results were reported as mean ± SD(^−^X ± S), range and percentage (%).

## Results

Among these patients, 36 (43%) were males and 47 (57%) were females, 69 (83%) were classified as B2 type fractures, and 14 (17%) were B3 type fractures. The average follow-up time was 94.5 ± 41.3 months (range: 2 to 137 months). The average age of these patients at the time of their revision surgery was 63.9 ± 9.5 years (range: 42 to 84 years) (Table 2).

**Table 2:** Baseline characteristics of the study patients

Classification	B2	B3	Total
Number	69(83%)	14(17%)	83(100%)
Gender (female/male)	38/31	9/5	
Age (years)	63.4 ± 9.1	66.4 ± 11.3	63.9 ± 9.5
Stem before revision			
uncemented	47(57%)	7(8%)	54(65%)
cemented stem	22(27%)	7(8%)	29(35%)
Injury mechanism			
mild trauma	59(71%)	5(6%)	64(77%)
immense trauma	6(7%)	7(8%)	13(15%)
unknown cause	4(5%)	2(3%)	6(8%)
Comorbidities (principal)			
Cardiovascular diseases	27(33%)	7(8%)	34(41%)
Respiratory diseases	15(18%)	3(4%)	18(22%)
Endocrine diseases	9(11%)	2(3%)	11(14%)
Other	7(8%)	2(3%)	9(11%)
ASA1	5(6%)	0	5(6%)
ASA2	12(14%)	2(3%)	14(17%)
ASA3	32(39%)	7(8%)	39(47%)
ASA4	20(24%)	5(6%)	25(30%)
SF-36 (Pre)	28.6 ± 10.4	31.0 ± 12.3	29.0 ± 10.7

The patients with B2 and B3 underwent revision surgery, and their bone defects were supplemented with bone grafting. A total of 43 patients underwent intraoperative bone grafting. In the B2 group, 20 of them received ICA (impacted cancellous allograft), and 5 received ICA + CSA (cortical strut allograft). In the B3 group, all patients received ICA + CSA bone graft. 7 patients with B2 fracture underwent ETO due to difficulty in removing original stem, and none of B3 received ETO. Among these patients, 80 (96.4%) achieved clinical healing at an average of 4.1 ± 0.6 months (range: 3 to 5 months), 2 (2.4%) developed non-union, and 1 (1.2%) died in the second month following the operation.

**Fig. 3:**
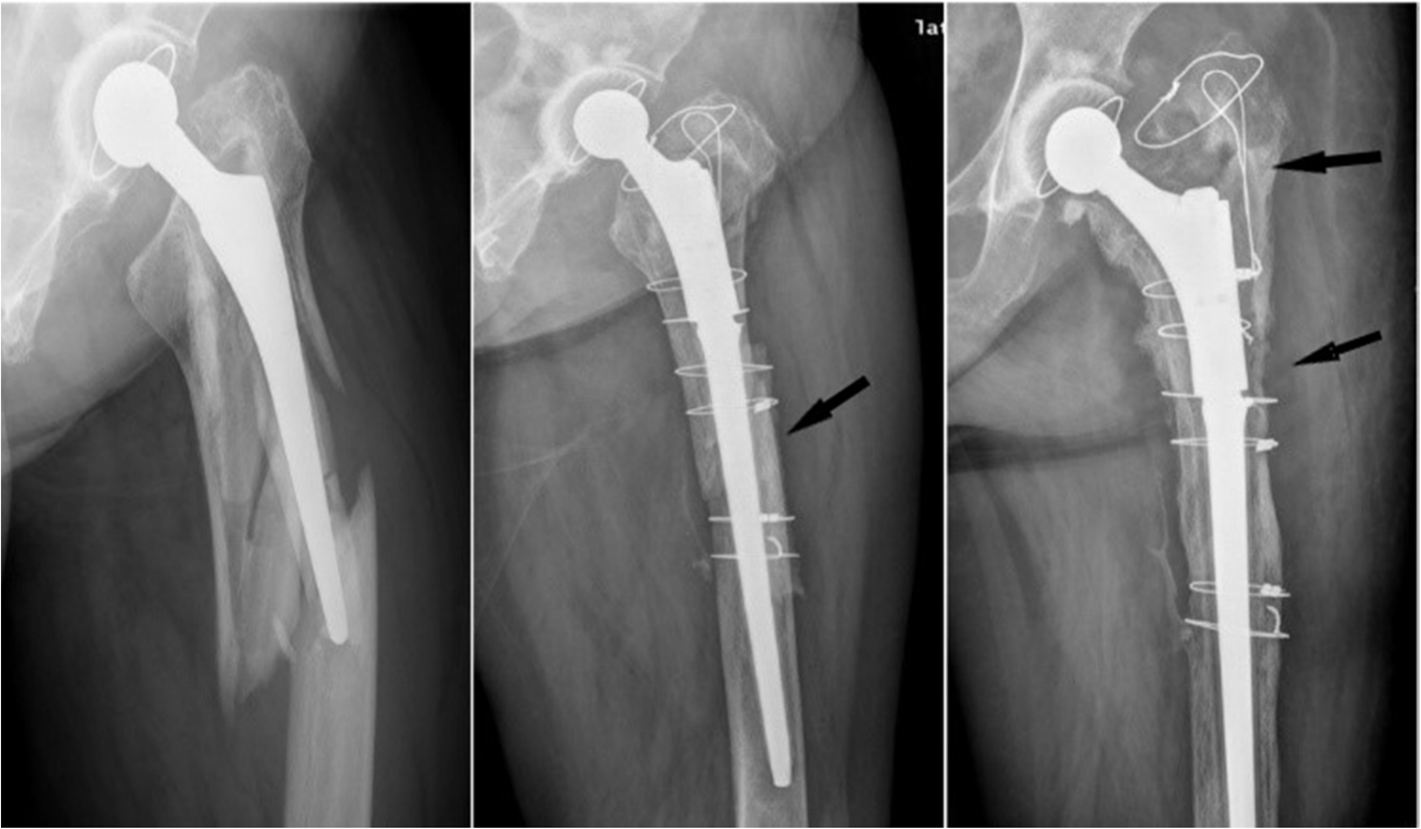
Uncemented stem, traffic accident, B3 fracture. Treated with ORIF + cortical strut allograft. In the third year after surgery, the prosthesis was significantly loosened and the patient received a second revision operation

Complications before discharge included 4 cases of incision complications, 2 cases of anemia, and 2 cases of urinary tract infection in B2 type PFFs. 1 cases of incision complications, 2 cases of anemia in B3 type PFFs. A total of 25 serious complications occurred during the 10-year follow-up, including 13 prosthetic dislocations, 8 aseptic loosening (Fig. 3), 2 infections, and 2 non-union (Table 3). Among these cases, 15 underwent secondary revision surgery with a revision rate of 18.1%. Prosthesis dislocation was identified as the primary cause of secondary revision.

**Table 3:** Serious complications which occurred during 10 year follow-up

complication	Number	Treatment
Dislocation	13	**7 B2 + 1 B3** recurrent dislocation revised to constrained liner
		**4 B2 + 1 B3:** closed reduction. No further dislocation
Aseptic loosening	8	**3 B2:** Conservative treatment and reduced activity
		**2 B2 + 3 B3:** Receive a second revision operation and allogeneic bone implantation
Infection	2	**1 B2:** Irrigation and debridement with head and liner exchange
		**1 B2:** Chronic antibiotic suppression
Nonunion	2	**1 B2:** Receive a second revision operation
		**1 B3:** Conservative treatment and reduced activity

A total of 8 patients underwent secondary revision surgery due to dislocation. 6 patients underwent a complete revision of the prosthetic stem and shell due to the small anterior angle of the cup and the sinking of the prosthetic stem. During the revision surgery, the position of the anteversion angle was adjusted, and a larger shell, matching femoral head prosthesis and stem were replaced. 2 cases of prosthetic stems were sinking, but the acetabular prosthesis was stable and in good position. We retained the shell and revised the prosthetic stem to a longer neck stem. During all revision surgery, none of the patients received dual mobility.

Those patients with PFFs demonstrated a high postoperative mortality. The highest mortality rate was recorded during the first year after operation (8.4%, 7/83). The Kaplan–Meier analysis results showed that the mortality rate tends to plateau after five years.

The 5-year Kaplan–Meier survival rates of patients with B2 and B3 fractures were 78.3% (95% CI, 68.5–88.1) and 64.3% (95% CI, 39.2–89.1), respectively. The ten-year survival rates were 67.1% (95% CI, 55.5–78.7) and 50.0% (95% CI, 23.8–76.3) respectively. The 5-year Kaplan–Meier survival rates for the implants with B2 and B3 fractures were 87.6% (95% CI, 79.0–96.2) and 83.3% (95% CI, 62.1–99.1), respectively. The ten-year survival rates were 81.9% (95% CI, 72.1–91.7) and 71.4% (95% CI, 44.2–99.6) respectively.

**Fig. 4:**
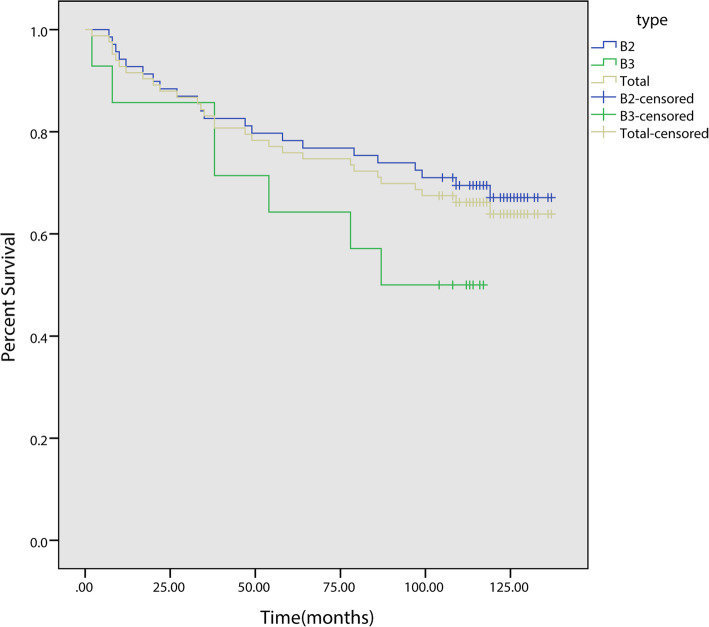
Kaplan-Meier survival curve for patients with B2 and B3 type PFFs after revision surgery

**Fig. 5:**
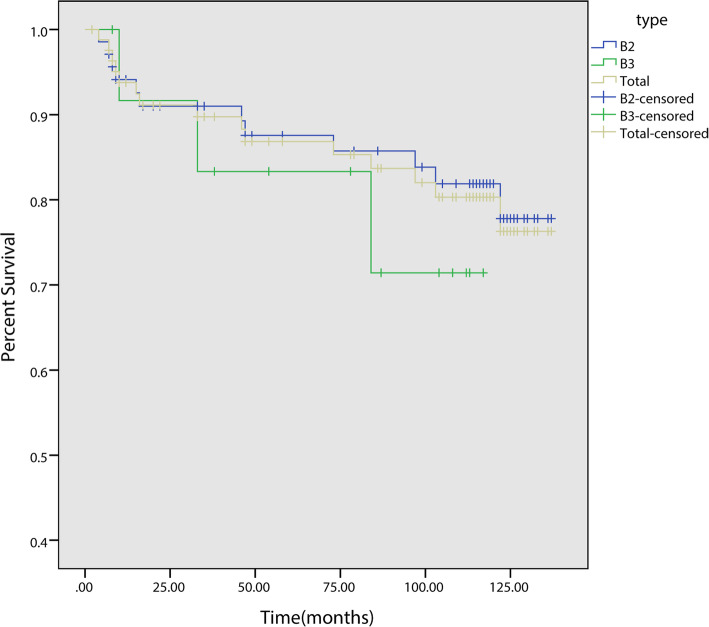
Kaplan-Meier survival curve (revision for any cause) of implants for patients with B2 and B3 type PFFs after revision surgery

**Fig. 6:**
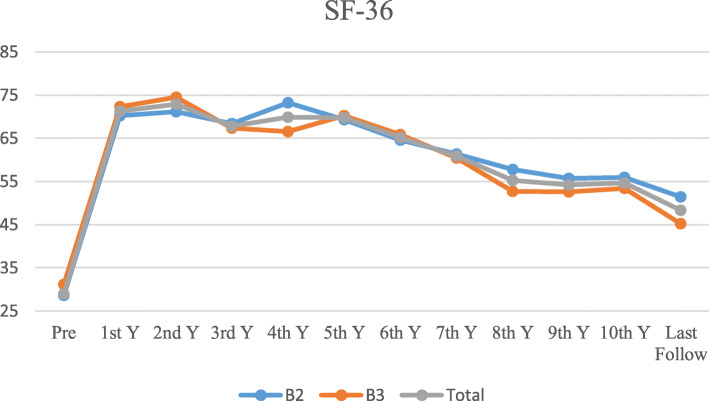
SF-36 score curve of patients with B2 and B3 fractures

The overall 5-year Kaplan–Meier survival rate for these patients was 75.9% (95% CI, 66.7–85.1), whereas the 10-year survival rate was 63.9% (95% CI, 53.1–74.7) (Fig. 4). Meanwhile, the 5-year Kaplan–Meier survival rate for the implants was 86.9% (95% CI, 79.3–94.5), whereas the 10-year survival rate was 80.3% (95% CI, 70.9–89.7) (Fig. 5). The survival curves for patients (*p* = 0.14) and implants (*p* = 0.47) showed no significant differences regardless of treatment type (distal fixation stem alone vs. distal fixation stem supplemented with cortical allograft). At the latest follow-up, the SF-36 score ranged from 17 to 64 with an average of 48.3 ± 9.8 (Fig. 6).

## Discussion

Early in the study of PFFs treatment, scholars tried to treat B2 or B3 PFFs conservatively. However, the results of the conservative treatment of patients with PFFs were very poor, and a very high incidence of complications, including atelectasis, thrombus, and non-union of fracture, was reported [11]. Surgical treatment was applied to allow patients to exercise early and to avoid complications resulting from conservative treatment. Despite showing improvements, the efficacy of surgical treatment remained far from satisfactory. The incidence of surgical complications and risk of death in PFFs surgery were considerably higher than those in aseptic loose hip revision surgery [12, 13].

The Kaplan–Meier survival analysis results showed a 5-year survival rate of 75.9% (95% CI, 66.7–85.1) and a 10-year survival rate of 63.9% (95% CI, 53.1–74.7), which were consistent with the data from the Swedish National Arthroplasty Registry (10-year rate: 64.9%) [14]. Compared with B3 type fractures, patients with B2 type fractures show higher survival rate and implants retention rate. This may be related to the worse bone condition and the higher difficulty during surgery of patients with B3 fractures.

The post-operative deaths among patients mostly occurred in the first year after surgery, with the earliest death reported in the second month after surgery. A mortality rate of 8.4% (7/83) was recorded within one year after surgery. Among patients of the similar age, the mortality rate of those patients with PFFs was significantly higher than that of patients with femoral neck fracture but was nearly similar to that of patients with hip fracture [15, 16]. Meanwhile, the Swedish National Arthroplasty Registry reported a 13.1% mortality rate among patients with PFFs during the first year after their surgery, and this rate agreed with the findings of Fuchtmeier [17], who reported a 13.2% mortality rate among patients within one year after surgery. Compared with the findings of this study, the data from Fuchtmeier better matched those from the Swedish National Arthroplasty Registry possibly due to the small number of research cases and subjects utilized in this study. In addition, the causes of short-term deaths after surgery were examined in this study (Table 4).

**Table 4:** Data of patients who died within one year after operation

Number	Time (month)	Cause of death
1	2	Multiple organ failure
2	7	Cerebral infarction
3	8	pulmonary embolism
4	8	Unknown cause of death
5	9	Renal failure
6	10	Multiple organ failure
7	12	pulmonary infection

The age, sex, Vancouver classification, surgical methods, operation time, and intraoperative blood loss of those seven patients who died within one year after their surgery were compared with those of other patients, and no statistically significant differences were observed in terms of sex, Vancouver classification, surgical methods, operation time, and intraoperative blood loss (*P* > 0.05). However, the age of these 7 patients was significantly higher than those of the other patients (*p* < 0.05). These patients, which included 5 females and 2 males, had an average age of 69.9 ± 3.9 years. Among them, three developed cardiovascular and cerebrovascular diseases before surgery, two had cardiovascular, cerebrovascular, and respiratory diseases, and two had cardiovascular and cerebrovascular diseases in addition to diabetes. The earliest post-operative death occurred in the second month after surgery. This patient developed persistent hypovolemic shock after surgery and eventually died of multiple organ failure. The other six patients were discharged from the hospital within two weeks after their surgery and returned to the local hospital for rehabilitation. The telephone follow-up revealed that almost all these patients were left bedridden for a long period after their surgery, thereby suggesting that long-term bedrest after surgery increases the risk of death among elderly patients.

Among the 15 patients who underwent secondary revision surgery, 8 had dislocated prosthesis, 5 had aseptic loosening, 1 had joint infection, and 1 had non-union. The revision rate among these patients was 18.1%, which was similar to that reported by the Swedish Joint Registry (18.7%). Mukundan [4] examined the surgical treatment outcomes of 59 patients with B2 and B3 PFFs for 2 years. Among these patients, 12 (20%) developed complications, including non-union, loosening, and prosthesis dislocation, and underwent second revision surgery. However, in this study, only 7 (8.4%) patients underwent a second revision during the 2-year follow-up, and this number is much lower than that reported by Mukundan. This result may be ascribed to the fact that the percentage of patients with B3 fractures in Mukundan’s work (28.8%) was much higher than that reported in this study (16.9%). In the B2 group, 11 patients underwent a second revision operation, 63.6% (7/11) of which was due to prosthesis dislocation. In the B3 group, 4 patients underwent a second revision operation, 75% (3/4) of which was due to Aseptic loosening. Patients with B3 fractures generally showed higher revision rates compared with the other patients. (28.6% vs 15.9%). However, the main reasons for revision were different between the two groups. Therefore, we recommend that in revision surgery of PFFs. For patients with type B2 fractures, we should pay more attention to the position of the prosthesis and the balance of soft tissue, while for type B3 fractures, we should focus more on bone mass and prosthesis stability.

Joint dislocation is a common complication resulting from a hip replacement that has a high chance of occurring during revision surgery [18]. In general, joint dislocation was identified as the main cause of secondary revision in this study (53.3%, 8/15), we believe that because multiple surgical operations can destroy the original anatomical structure of the hip and result in a poor soft tissue balance.

The prosthesis mainly subsided within 1 year after surgery, which coincided with the findings of Mulay [19], who found that the prosthesis mainly subsided within 6 months after revision with an average subsidence of 5 mm. However, the prosthesis did not subside again during the five-year follow-up after bony ingrowth and fracture healing were achieved.

SF-36 was used to assess the quality of life of patients. SF-36 is a general health assessment tool that comprehensively evaluates the quality of life of patients from eight aspects, including bodily functions, social functions, and mental health.

This work has several limitations. First, this is a retrospective study, only a few cases were examined, and additional sample data are warranted. Second, the stems, surgical methods, bone grafting methods, and postoperative rehabilitation training methods in revision surgery are not unified, which may cause bias. Third, the classification of fractures is determined by the surgeon pre-and intra-operation, this may be subjective. Finally, this study was conducted by two hospitals whose surgeons may have different surgical experiences.

## Conclusions

Patients with fractures around the femoral prosthesis have a high mortality rate, especially during the first year after their surgery. The Kaplan–Meier analysis revealed that this mortality rate tends to plateau after five years. Prosthesis dislocation was identified as the primary cause of secondary revision.

## Data Availability

All data generated or analyzed during this study are included in this article.

## Abbreviation

PFFs: Periprosthetic femoral fractures
